# Supplementary material for: 5mC modification orchestrates choriogenesis and fertilization by preventing prolonged ftz-f1 expression
Source: Nat Commun. 2023 Dec 12;14:8234. doi: 10.1038/s41467-023-43987-5 (PMC10716119; doi:10.1038/s41467-023-43987-5)

Supplementary Information for

5mC modification orchestrates choriogenesis and fertilization by preventing prolonged *ftz-f1* expression

Zheng Zhao[1, 2, 3, †], Liang Li[1, †], Ruichen Zeng[1], Liangguan Lin[1], Dongwei Yuan[1], Yejie Wen[1], Na Li[1, 3], Yingying Cui[1], Shiming Zhu[1], Zhi-Min Zhang[4], Sheng Li[1, 2, 3, *], and Chonghua Ren[1, 3, *]

[1]Guangdong Provincial Key Laboratory of Insect Developmental Biology and Applied Technology, Institute of Insect Science and Technology & School of Life Sciences, South China Normal University, Guangzhou 510631, China

[2]Guangdong Laboratory for Lingnan Modern Agriculture, Guangzhou 510631, China

[3]Guangmeiyuan R&D Center, Guangdong Provincial Key Laboratory of Insect Developmental Biology and Applied Technology, South China Normal University, Meizhou 514779, China

[4]College of Pharmacy, Jinan University, 510632, Guangzhou, China.

†These authors contributed equally to this work.

*Corresponding authors. Sheng Li and Chonghua Ren
Email: lisheng@scnu.edu.cn; renchonghua111@m.scnu.edu.cn

Supplementary figures and figure legends

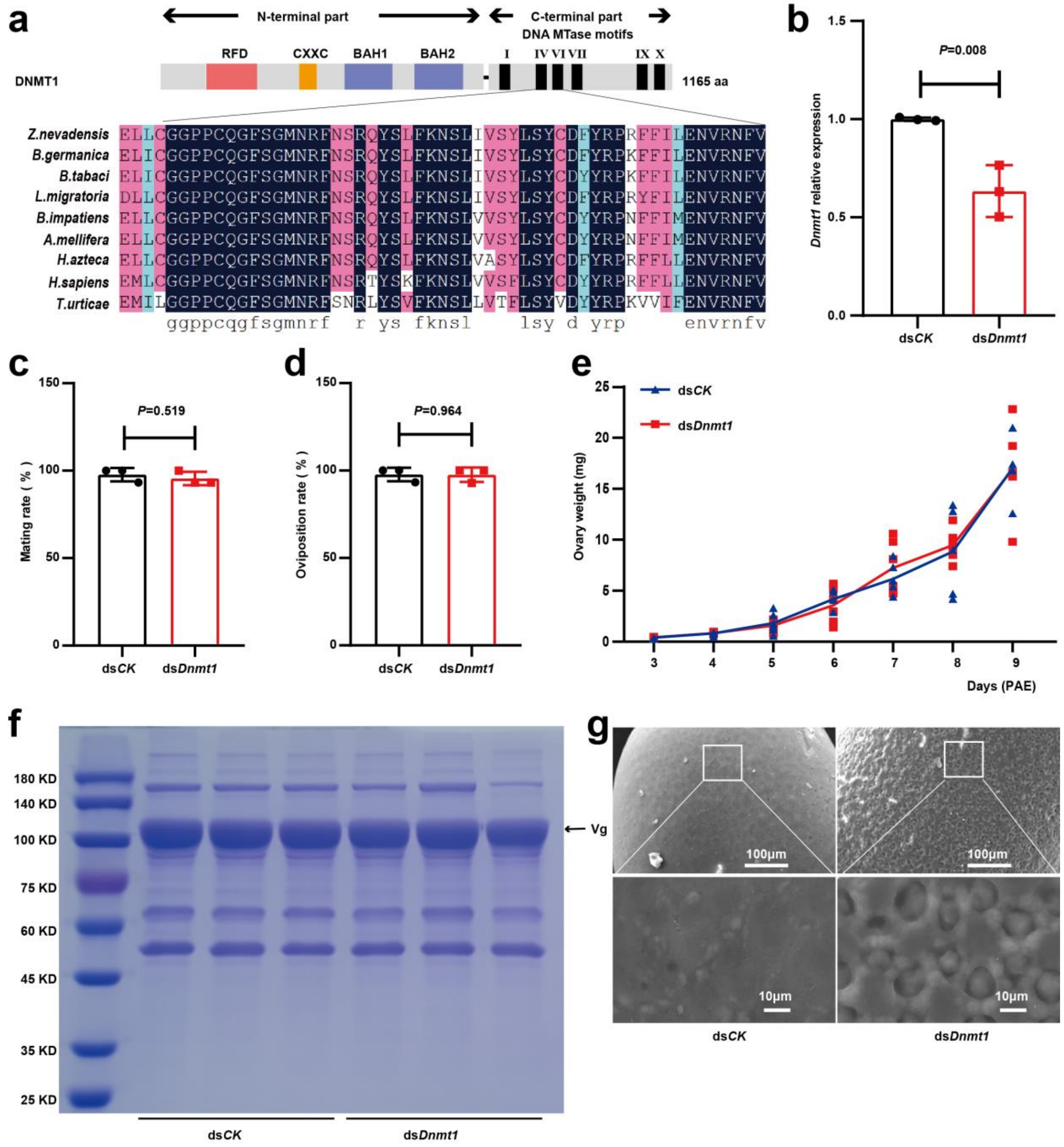

Fig. S1: Functional studies on the roles of DNMTs in 5mC modification in female adults. (a). Evolutionary conservation analyses of DNMT1s in several insects and human. (b). RNAi efficiency detection for *Dnmt1*. Data are mean±sd, the differences were analysed by two-tailed Student's *t* test, n=3 biologically independent samples. (c). Statistics of mating rate under ds*Dnmt1* treatment. Data are mean±sd, the differences were analysed by two-tailed Student's *t* test, n=3 biologically independent. (d). Statistics of oviposition rate under ds*Dnmt1* treatment. Data are mean±sd, the differences were analysed by two-tailed Student's *t* test, n=3 biologically independent experiments. (e). Statistics of ovary weight under ds*Dnmt1* treatment. Data are mean±sd, n=5 biologically independent samples. (f). Ovarian whole-protein staining using Coomassie blue reagent, the main component Vg protein in the oocytes was marked. (g). SEM observation of the ovarian surface (tunica propria). The differences were analysed by two-tailed Student's *t* test. Source data are provided as a Source Data file.

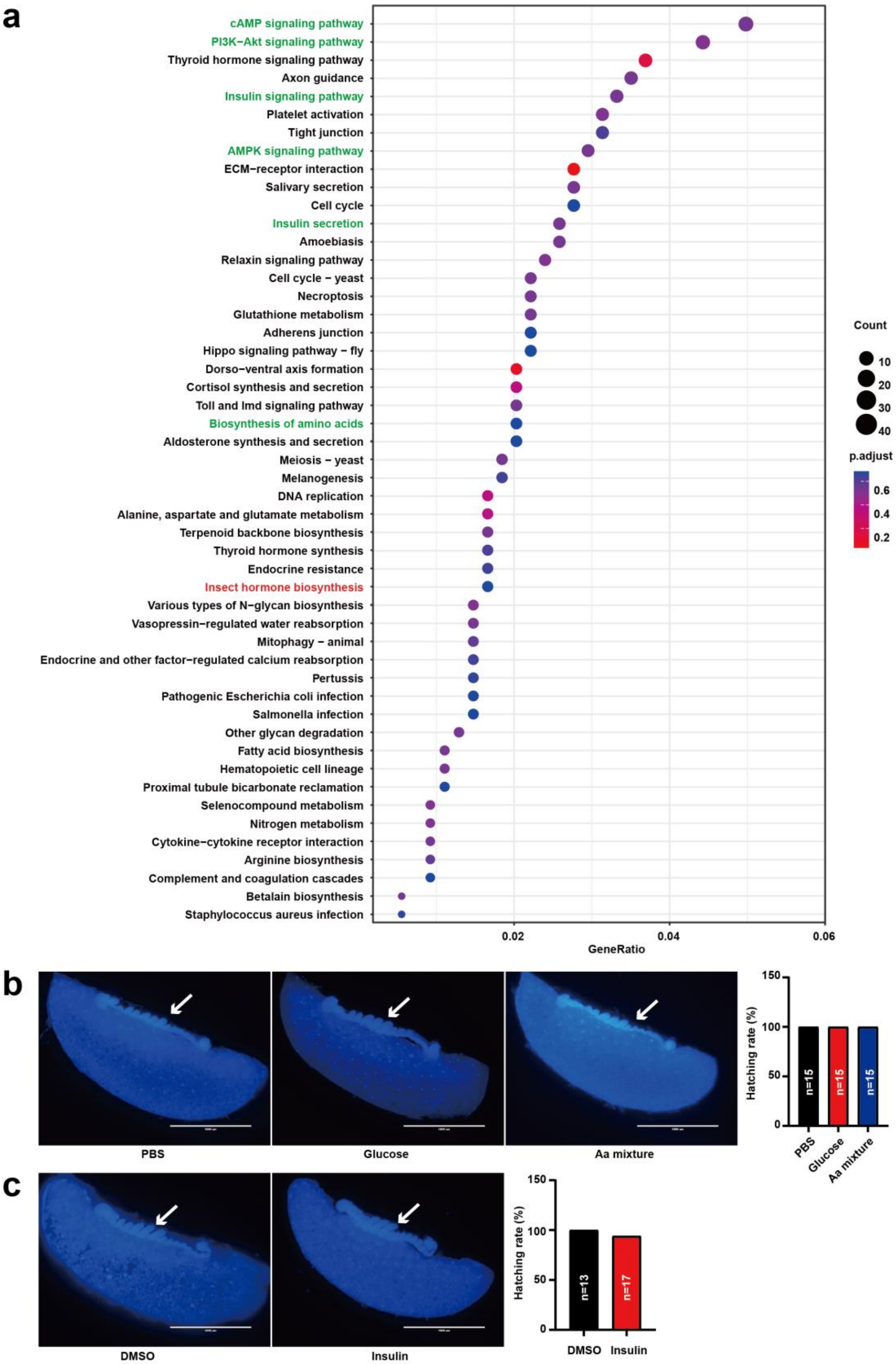

Fig. S2: Screening of candidate pathways involved in *Dnmt1*-controlled fertilization. (a). KEGG analysis of upregulated DEGs. Both nutrition related (green labeled) and insect hormone biosynthesis (magenta labeled) pathways were found being enriched on day 9 PAE between the ds*Dnmt1*and ds*CK* groups. (b-c). Neither exogenous glucose, Aa mixture (b) nor insulin (c) treatments affect hatching rate significantly. The arrows point to the position of embryogenesis. 6 eggs were observed in each treatment group. n means biologically independent animals. Source data are provided as a Source Data file.

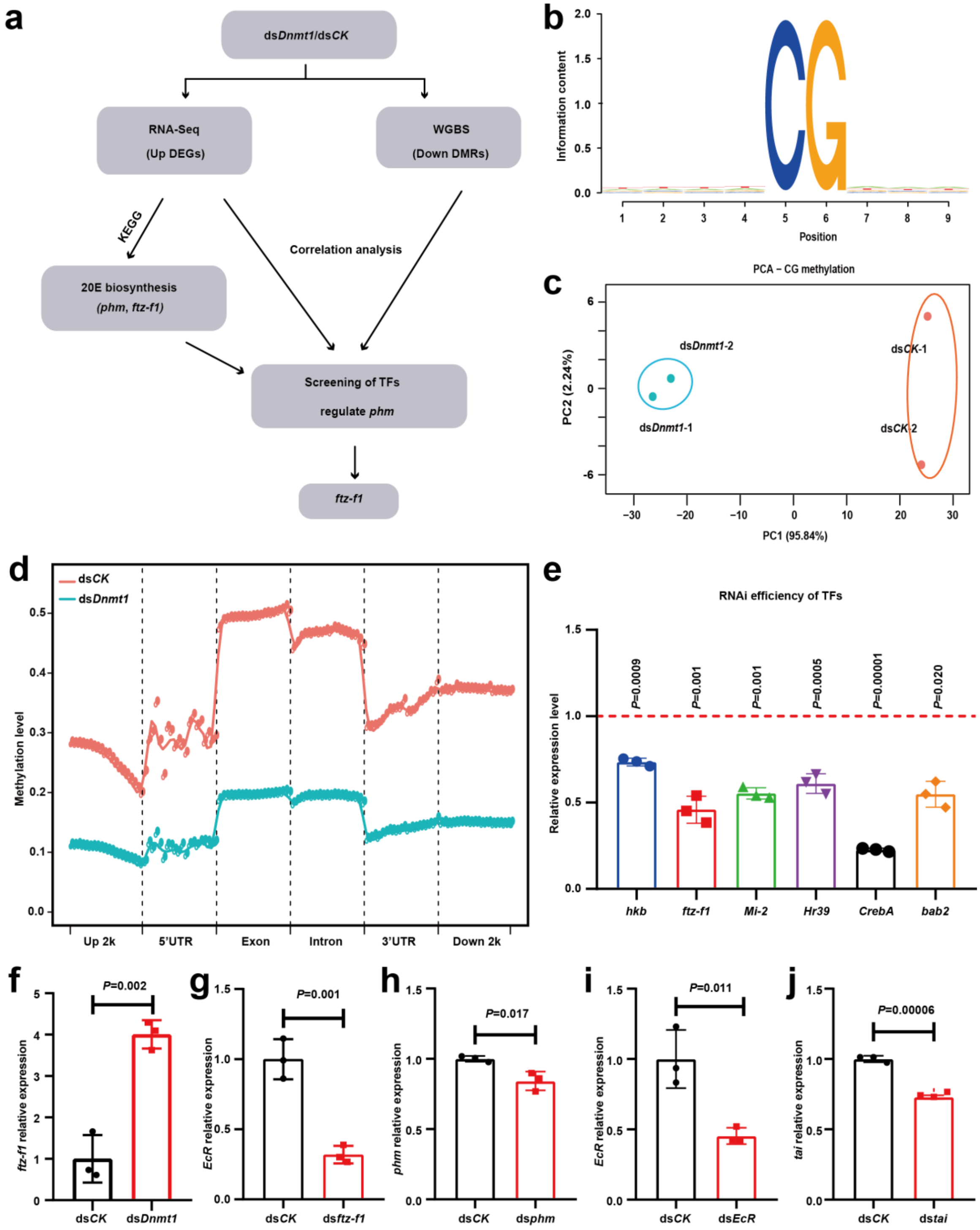

Fig. S3: The screening of *ftz-f1* which regulates steroidogenesis. (a). The flowchart of screening of *ftz-f1* from RNA-Seq and WGBS data. (b). seqLogo motif results for methylation motifs in the genome of *B. germanica*. (c). PCA of gene methylation changes between ds*CK* and ds*Dnmt1* at day 9 PAE. n= 2 biologically independent samples. (d). Average 5mC levels in different functional gene regions of the genome, including the Up 2k, 5'UTR, exons, introns, 3'UTR and downstream 2 kb. (e). RNAi efficiency of top six TF genes with >2-fold upregulation detected by qRT-PCR. (f). *ftz-f1* relative expression level under ds*Dnmt1* treatment detected by qRT-PCR. (g). Relative expression of *EcR* when *ftz-f1* was knocked down detected by qRT-PCR. (h-j). RNAi efficiency of *phm*, *EcR*, and *tai* detected by qRT-PCR. Data are mean ±sd, n=3 biologically independent samples (e-j). The differences were analysed by two-tailed Student's *t* test. Source data are provided as a Source Data file.

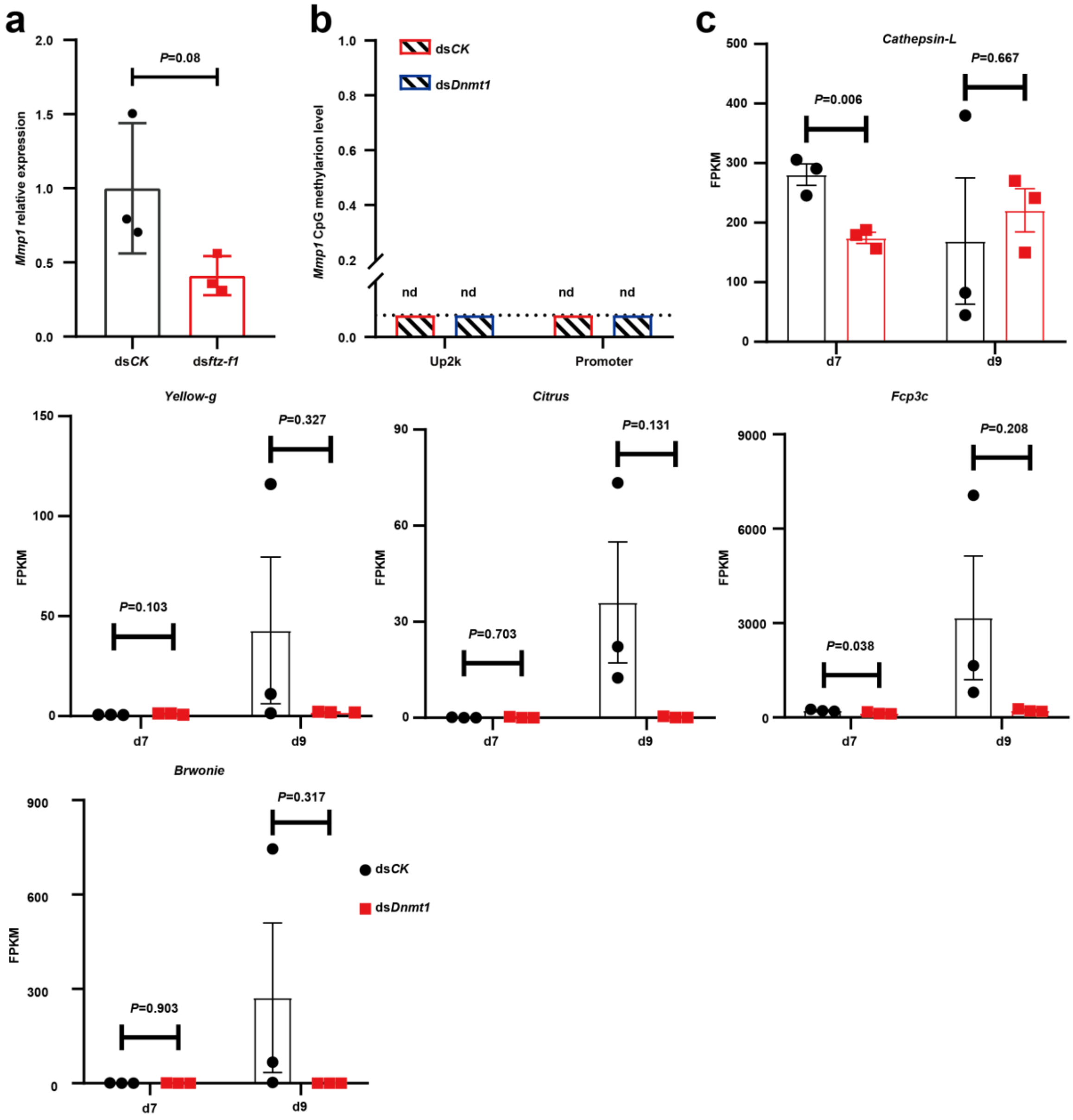

Fig. S4: Statistics of gene methylation levels and expression levels. (a). The gene expression level of *Mmp1* under *ftz-f1* knock down treatment detected by qRT-PCR, data are mean±sd, n=3 biologically independent samples. (b). Methylation levels of the *Mmp1* in the Up 2k and promoter regions. “nd” stands for “not detectable”. Data are mean±sd, n= 2 biologically independent samples. (c). The expression levels (FPKM) of chorion genes at both d7 and d9 PAE under dsRNA treatments, data are mean±sem, n=3 biologically independent samples. All the differences were analysed by two-tailed Student’s *t* test. Source data are provided as a Source Data file.

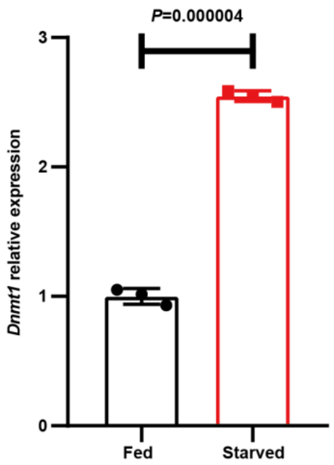

Fig. S5: *Dnmt1* relative expression level under food deprivation treatment detected by qRT-PCR. Data are mean ±sd, n=3 biologically independent samples, the differences were analysed by two-tailed Student's *t* test. Source data are provided as a Source Data file.

Table S1: qPCR primers for gene expression detection

Primer names	Primer sequence (5' →3')
Actin-qRT-F	TGAGACCACATACAACTCCA
Actin-qRT-R	CAATTCCAGGGTACATGGTGGTG
Dnmt1-qRT-F	ACTGGAAAAGCGAGACATTC
Dnmt1-qRT-R	GATGCAGTCG TTCTTTCGTATC
ftz-f1-qRT-F	CCATATTGCCGCTTCCA
ftz-f1-qRT-R	GGGCTCTGTCCCTCTTGTA
Mmp1-qRT-F	GTGTGGTCGCAGGTTTCG
Mmp1-qRT-R	CCGTGCTCTCCCTTCTCG
tai-qRT-F	CAACCTCCGTCCACAACGCAAAG
tai-qRT-R	GCTTGCTGCTGCTGTTGAAGTTG
phm-qRT-F	GTTTCATCATCTGCTCGCA
phm-qRT-R	CCATTTCATCCTGTACTCTCTTC
EcR-qRT-F	GTCTCCGGAAGTAGTGATGA
EcR-qRT-R	ATTCACTGTCGGTAGCTTCT
Brownie-qRT-F	ATGATTCTAGTCGTCTCGCT
Brownie-qRT-R	TCCTCCATAAAGCCACTCAT
Fcp3c-qRT-F	TTTCGTAGCAGCGATGTTC
Fcp3c-qRT-R	TGCTGATTAGAGGTGGTAGAGTA
Yellow-g-qRT-F	CTCATCCCTCAGTAAAGACCA
Yellow-g-qRT-R	GAACACCCTCCTTCAATCG
Citrus-qRT-F	TTACCCACGGATACCCATAC
Citrus-qRT-R	TTTCCATGGGTGACCAAAAG
Cathepsin L-qRT-F	TCCTGTGTCTGTGGCAATAG
Cathepsin L-qRT-R	ACCAGCACTCCATGATCAAG
bab2 qRT-F	TAGCAGTGCTGGAGAAGTTG
bab2 qRT-R	CCTGGTGCTTGCATATAAGG

CrebA-qRT-F	CCCAAGTAGCGAGAGTAGTC
CrebA-qRT-R	GCCTCGTCTTCCTTGGATTC
hkb-qRT-F	TTTGGTGGTGCTGGTCTAGG
hkb-qRT-R	ACCACTTATGGGACCAACTG
Hr39-qRT-F	AAATCTGCGGAGTGAATGCC
Hr39-qRT-R	AATGGTCCCGTTTGTGGATG
Mi-2-qRT-F	TCATCCGTACCAGTTGGAAG
Mi-2-qRT-R	CGTATCACAGCTCTCGAATC

Table S2: Primers for dsRNA synthesis

Primers names	Primer sequence (5'→3')
Dnmt1-dsRNA-F	**GGATCCTAATACGACTCACTATAGG**GAGTGCGTGGTCAACTTTAG
Dnmt1-dsRNA-R	**GGATCCTAATACGACTCACTATAGG**TGAACGCATGAGCGGCGGA
ftz-f1-dsRNA-F	**GGATCCTAATACGACTCACTATAGG**TGCGAGTCCTGCAAAGGATTCTTC
ftz-f1-dsRNA-R	**GGATCCTAATACGACTCACTATAGG**CGATTATGTGGAGAGTGCCCTGCT
phm-dsRNA-F	**GGATCCTAATACGACTCACTATAGG**GATCTACAGCCTCAGAATGGGCAG
phm-dsRNA-R	**GGATCCTAATACGACTCACTATAGG**GAATGTGCTGCAGCCATTTCCAAG
tai-dsRNA-F	**GGATCCTAATACGACTCACTATAGG**AAAGGACTTCTGAATCAGGATGACA
tai-dsRNA-R	**GGATCCTAATACGACTCACTATAGG**AATGGGAGGAATAGTTGCAGTTTTG
EcR-dsRNA-F	**GGATCCTAATACGACTCACTATAGG**ACCTTCAGAAGAGGACCTCAA
EcR -dsRNA-R	**GGATCCTAATACGACTCACTATAGG**CGGTTGTCTACATACGCCTT
bab2-dsRNA-F	**GGATCCTAATACGACTCACTATAGG**GTTGGCAGCTGAAGGAAAGC
bab2-dsRNA-R	**GGATCCTAATACGACTCACTATAGG**TGCACCCCATGGAGATTCAC
CrebA-dsRNA-F	**GGATCCTAATACGACTCACTATAGG**GAGCAACCACTCTCCAGACC
CrebA-dsRNA-R	**GGATCCTAATACGACTCACTATAGG**CCAGCACCACTTGCAACTTC
hkb-dsRNA-F	**GGATCCTAATACGACTCACTATAGG**AGGTGCCAAGTCTGAGAGGA
hkb-dsRNA-R	**GGATCCTAATACGACTCACTATAGG**TGAATGCGAAGATGCCGAGT
Hr39-dsRNA-F	**GGATCCTAATACGACTCACTATAGG**CATCAATGTCCAGCATGGCG
Hr39-dsRNA-R	**GGATCCTAATACGACTCACTATAGG**CCTGCGAATCCTCACAACCT
Mi-2-dsRNA-F	**GGATCCTAATACGACTCACTATAGG**GAGCAAACCTGTGACTCCGA
Mi-2-dsRNA-R	**GGATCCTAATACGACTCACTATAGG**GGTCCTGCGTCAAGTTCAGA

Note: All the primers in Table S2 with T7 promoter sequences "GGATCCTAATACGAC TCACTATAGG" at their 5' ends for dsRNA synthesis.

Table S3: NGS-BSP primers for *ftz-f1*

Primers names	Primer sequence (5'→3')
ftz-f1-BSP-F1	GTGATTATAATTAGGTTTTGATGAAAATTTAGTAA
ftz-f1-BSP-R1	CACTACAAAACCAAATATAAAATAAAATCATTCAT
ftz-f1-BSP-F2	YGGTGTTTGGGGGATAATTTTTAAATAT
ftz-f1-BSP-R2	CATTATTCTACTTCTACTATATTTTATATTATCTTAC
ftz-f1-BSP-F3	TGTAGTATTGTAGTAGGTTAATATTTAGG
ftz-f1-BSP-R3	CAAAACRTAACAATAACACATTTTAATTTTATTATC
ftz-f1-BSP-F4	GTTATAAGAAAATGAAATTTATTATTGGTTAGGAT
ftz-f1-BSP-R4	CACTATAAACAACCCATACTTTAATTATATTATC

ftz-f1-BSP-F5	TAYGTTTGAGAATGTTAAATAATGATTTAGGTTAT
ftz-f1-BSP-R5	CRAAAATCTATCCAATATAAATTAATTTAATAATCCC
ftz-f1-BSP-F6	ATTATTGAATATTTTAGAATAGYGATGTTAGTTAG
ftz-f1-BSP-R6	CTTCTAAAATCCAATTAACTTAAAATTTACTACC

For figure S1F

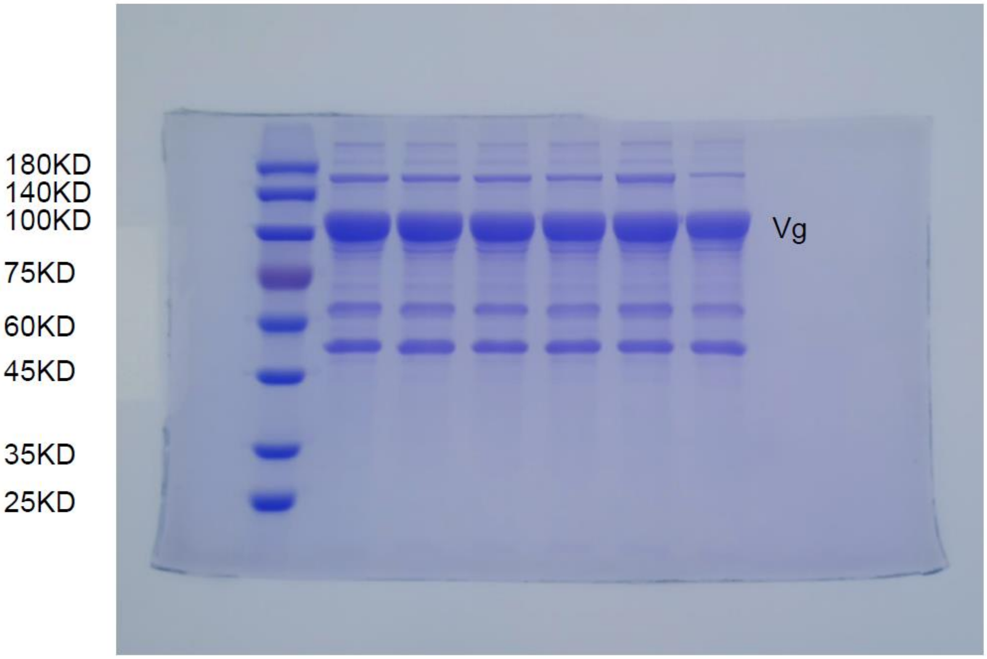

Supplement: Supplementary file 1 — Supplementary Information [file 41467_2023_43987_MOESM1_ESM.pdf]
