# Supplementary material for: 5mC modification orchestrates choriogenesis and fertilization by preventing prolonged ftz-f1 expression
Source: Nat Commun. 2023 Dec 12;14:8234. doi: 10.1038/s41467-023-43987-5 (PMC10716119; doi:10.1038/s41467-023-43987-5)
Supplement: Supplementary file 3 — Reporting Summary [file 41467_2023_43987_MOESM3_ESM.pdf]

nature portfolio

Corresponding author(s): Sheng LI, Chonghua Ren

Last updated by author(s): Nov 3, 2023

Reporting Summary

Nature Portfolio wishes to improve the reproducibility of the work that we publish. This form provides structure for consistency and transparency in reporting. For further information on Nature Portfolio policies, see our Editorial Policies and the Editorial Policy Checklist.

Statistics

For all statistical analyses, confirm that the following items are present in the figure legend, table legend, main text, or Methods section.

n/a	Confirmed	
☐	☒	The exact sample size (*n*) for each experimental group/condition, given as a discrete number and unit of measurement
☐	☒	A statement on whether measurements were taken from distinct samples or whether the same sample was measured repeatedly
☐	☒	The statistical test(s) used AND whether they are one- or two-sided<br>*Only common tests should be described solely by name; describe more complex techniques in the Methods section.*
☐	☒	A description of all covariates tested
☐	☒	A description of any assumptions or corrections, such as tests of normality and adjustment for multiple comparisons
☐	☒	A full description of the statistical parameters including central tendency (e.g. means) or other basic estimates (e.g. regression coefficient) AND variation (e.g. standard deviation) or associated estimates of uncertainty (e.g. confidence intervals)
☐	☒	For null hypothesis testing, the test statistic (e.g. *F*, *t*, *r*) with confidence intervals, effect sizes, degrees of freedom and *P* value noted<br>*Give P values as exact values whenever suitable.*
☒	☐	For Bayesian analysis, information on the choice of priors and Markov chain Monte Carlo settings
☒	☐	For hierarchical and complex designs, identification of the appropriate level for tests and full reporting of outcomes
☐	☒	Estimates of effect sizes (e.g. Cohen's *d*, Pearson's *r*), indicating how they were calculated

Our web collection on statistics for biologists contains articles on many of the points above.

Software and code

Policy information about availability of computer code

Data collection	No novel software was used in this work.
Data analysis	No novel software was used in this work.

For manuscripts utilizing custom algorithms or software that are central to the research but not yet described in published literature, software must be made available to editors and reviewers. We strongly encourage code deposition in a community repository (e.g. GitHub). See the Nature Portfolio guidelines for submitting code & software for further information.

Data

Policy information about availability of data

All manuscripts must include a data availability statement. This statement should provide the following information, where applicable:

- Accession codes, unique identifiers, or web links for publicly available datasets
- A description of any restrictions on data availability
- For clinical datasets or third party data, please ensure that the statement adheres to our policy

All data and materials to understand and assess the conclusions of this research are available in the main text, supplementary materials, and via the Sequence Read Archive (SRA) database under accession numbers PRJNA700816.

Research involving human participants, their data, or biological material

Policy information about studies with human participants or human data. See also policy information about sex, gender (identity/presentation), and sexual orientation and race, ethnicity and racism.

Reporting on sex and gender	Not mentioned.
Reporting on race, ethnicity, or other socially relevant groupings	Not mentioned.
Population characteristics	Not mentioned.
Recruitment	Not mentioned.
Ethics oversight	Not mentioned.

Note that full information on the approval of the study protocol must also be provided in the manuscript.

Field-specific reporting

Please select the one below that is the best fit for your research. If you are not sure, read the appropriate sections before making your selection.

- [x] Life sciences
- [ ] Behavioural & social sciences
- [ ] Ecological, evolutionary & environmental sciences

For a reference copy of the document with all sections, see nature.com/documents/nr-reporting-summary-flat.pdf

Life sciences study design

All studies must disclose on these points even when the disclosure is negative.

Sample size	Not less than three replicates were used in this study.
Data exclusions	No data exclusion in this study.
Replication	All attempts at replication are successful.
Randomization	All the samples were randomly allocated into different experimental groups.
Blinding	The investigators were blinded to group allocation during data collection and analysis.

Reporting for specific materials, systems and methods

We require information from authors about some types of materials, experimental systems and methods used in many studies. Here, indicate whether each material, system or method listed is relevant to your study. If you are not sure if a list item applies to your research, read the appropriate section before selecting a response.

Materials & experimental systems

n/a	Involved in the study	
☐	☒	Antibodies
☒	☐	Eukaryotic cell lines
☒	☐	Palaeontology and archaeology
☐	☒	Animals and other organisms
☒	☐	Clinical data
☒	☐	Dual use research of concern
☒	☐	Plants

Methods

n/a	Involved in the study	
☒	☐	ChIP-seq
☒	☐	Flow cytometry
☒	☐	MRI-based neuroimaging

Antibodies

Antibodies used	5mC-specific antibody (Abcam, 33D3); α-tubulin antibody (Sigma, MAB1864-I); Caspase 3 antibody (Beyotime, AC033)
Validation	These commercial antibodies are first used in the B. germanica species.

Animals and other research organisms

Policy information about studies involving animals; ARRIVE guidelines recommended for reporting animal research, and Sex and Gender in Research

Laboratory animals	Adult Blattella germanica (Linnaeus) were used in this study.
Wild animals	This study did not involve wild animals.
Reporting on sex	All the data were collected using adult females since we focused on the female insect reproduction.
Field-collected samples	To maintain the colony, cockroaches were reared in plastic jars at 28 °C and 70% relative humidity in the dark. They were provided with rat chow and water.
Ethics oversight	All animal protocols were reviewed and approved by the University Animal Care and Use Committee of South China Normal University.

Note that full information on the approval of the study protocol must also be provided in the manuscript.
