# Supplementary figures and images for: 5mC modification orchestrates choriogenesis and fertilization by preventing prolonged ftz-f1 expression
Source: Nat Commun. 2023 Dec 12;14:8234. doi: 10.1038/s41467-023-43987-5 (PMC10716119; doi:10.1038/s41467-023-43987-5)

For figure 1A

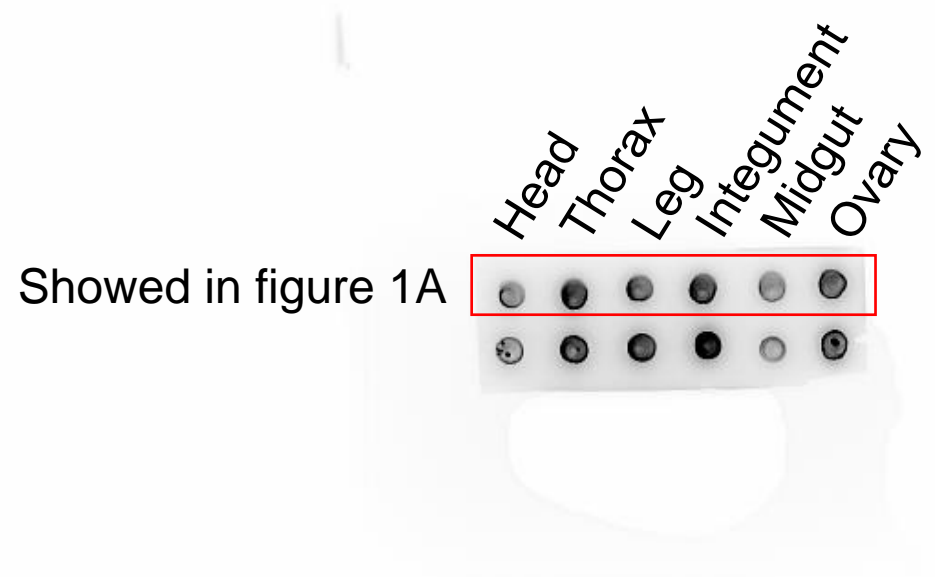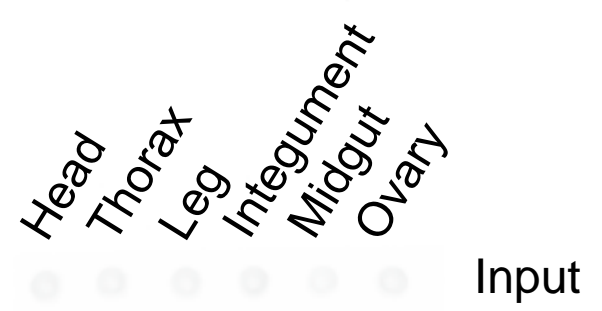

For figure 1A'

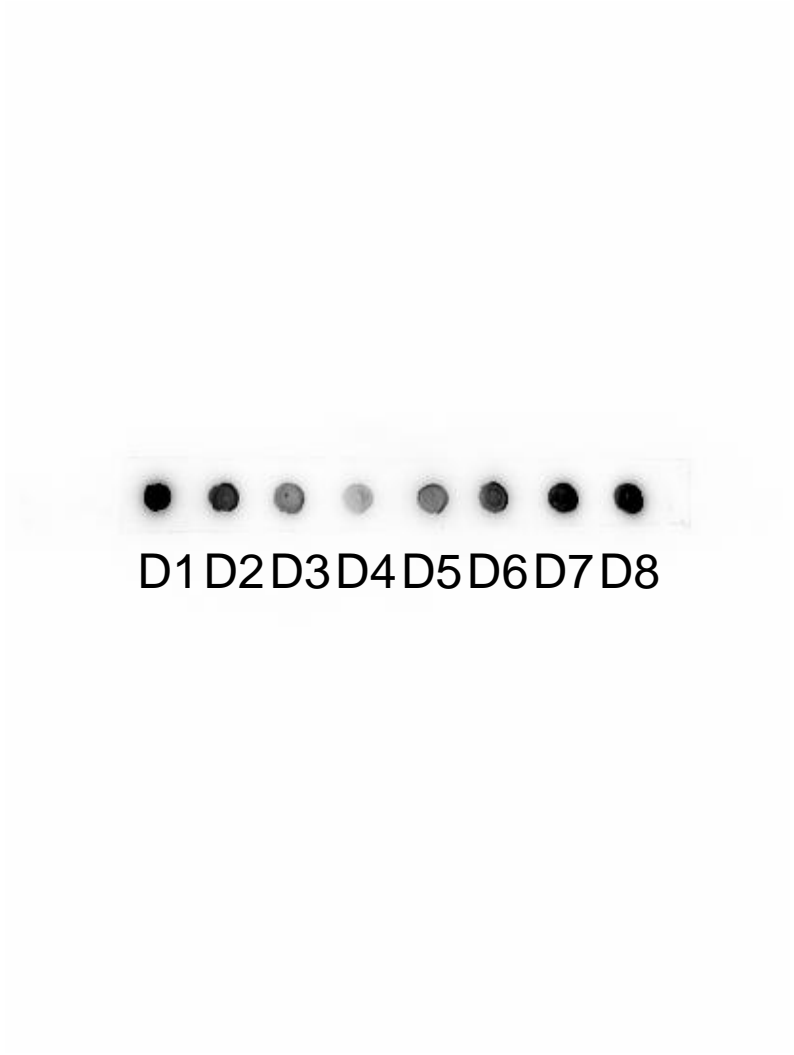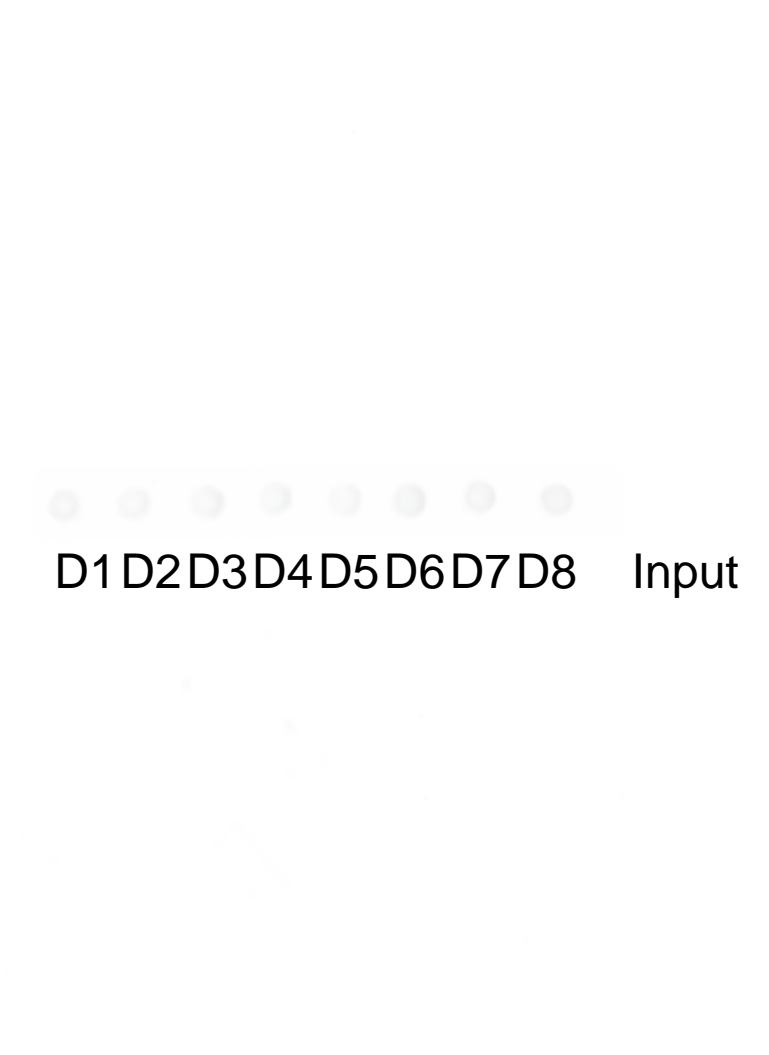

For figure 1C

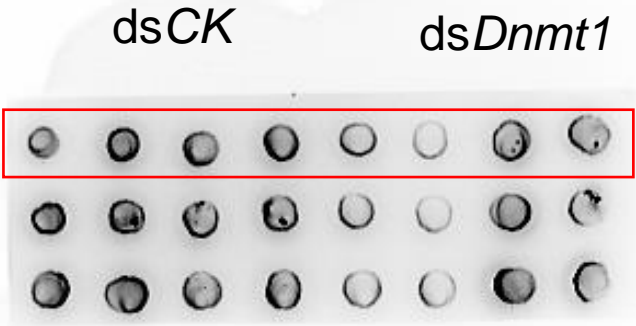

Showed in figure 1C

Supplement: Supplementary file 4 — Source Data [file 41467_2023_43987_MOESM4_ESM.zip › Original picture for dot bloting.pdf]
